# European Teas (*Camellia sinensis*) as a New Frontier in the Specialty Tea Market: Characterizing the Antioxidant, Polyphenolic, and Sensory Profiles Through a Systematic, Comparative Approach

**DOI:** 10.3390/antiox15010141

**Published:** 2026-01-22

**Authors:** Patricia Carloni, Benedetta Fanesi, Paolo Lucci, Cristina Truzzi, Federico Girolametti, Elisabetta Damiani

**Affiliations:** 1 Department of Agricultural, Food and Environmental Sciences-D3A, Università Politecnica delle Marche, Via Brecce Bianche, 60131 Ancona, Italy; p.carloni@staff.univpm.it (P.C.); b.fanesi@univpm.it (B.F.); p.lucci@staff.univpm.it (P.L.); 2 Department of Life and Environmental Sciences-DISVA, Università Politecnica delle Marche, Via Brecce Bianche, 60131 Ancona, Italy; c.truzzi@staff.univpm.it (C.T.); f.girolametti@staff.univpm.it (F.G.)

**Keywords:** *Camellia sinensis*, flavor profiles, European teas, polyphenolic profile, antioxidant activity, specialty tea market

## Abstract

Tea production in Europe represents an emerging segment of the specialty tea market, but a structured comparative analysis remains unexplored. This study employs a standardized approach to systematically characterize hot brews from black and green teas across five European gardens. Antioxidant capacity, total polyphenolic content (TPC), total flavonoid content (TFC), and metabolomic profiling by ultra-high performance liquid chromatography–mass spectrometry were evaluated, and for the first time, sensory profiling of these teas was conducted. Green teas consistently exhibited higher TPC, TFC, and antioxidant capacity compared to black teas, confirming the influence of processing methods. Metabolomic analysis revealed variability in caffeine linked to geographical origin and propagation method (cuttings vs. seeds). Importantly, sensory evaluation suggested a negative correlation between high TPC and overall consumer appreciation. The two most highly appreciated teas often showed lower TPC. These reliable findings advance knowledge in European tea research, providing valuable data for growers to enhance cultivar selection and marketing strategies in alignment with consumer preferences.

## 1. Introduction

Tea growing in Europe is relatively recent compared to traditional tea-growing regions like Asia and Africa. The beverage made from the tender leaves and buds of the tea plant, *Camellia sinensis* (L.), O. Kuntze, which is the most popular drink worldwide after water, was first introduced to Europe in the 16th century by Portuguese traders who had established trade routes to China. Its popularity spread across Europe through “The Dutch East India Company”, which began importing tea from China in the early 17th century [1]. It was only in the late 19th century on the Atlantic island of São Miguel in the Azores that the first successful tea plantations in Europe were established and where the oldest, continuously operating tea plantations in Europe are found [2]. However, in more recent years, other European countries in areas with a unique microclimate and soil that provide the ideal conditions for tea cultivation have followed suit, and in 2016 the Tea Grown in Europe Association (EuTA) was founded to promote the growing of tea as an economic activity. It supports its members in developing know-how in utilizing the best practices for environment-friendly cultivation, harvesting, and processing techniques, as well as for members to obtain national or European recognition labels and to protect vital botanic varieties relevant to the cultivation of tea in Europe, collected by the members [3]. Hence, these new tea gardens are contributing to the diversity and specialty tea market in Europe, which reflects a blend of historical trade influences and modern agricultural innovation, offering unique flavors and qualities distinct from traditional tea-growing regions.

Nevertheless, the characterization of European tea remained unexplored until 2023, when we began to study the health-promoting properties of both cold and hot brews from green, black, and white teas in terms of antioxidant capacity, polyphenolic/flavonoid content, and metal chelating activity from tea plants grown in 7 different European countries, members of the EuTA [4,5]. Metabolomic profiling was also conducted, but only hot, green tea infusions. Additionally, chemical fingerprinting of the tea leaves and of their corresponding hot and cold brews was obtained using ATR-FTIR (Attenuated Total Reflectance Fourier Transform InfraRed) spectroscopy, combined with chemometrics for further characterization [6].

However, in all these studies, standardizing the sampling and processing of the tea leaves in a systematic manner for comparative purposes had not been carried out. This is fundamental for several reasons. Standardization ensures uniform collection and processing of samples, facilitating easier comparison of results across different tea-growing regions. This crucial aspect is often overlooked in most comparative tea studies conducted worldwide. Uniform, consistent methods ensure that all samples meet the same criteria, thus reducing variability caused by different sampling and processing techniques. This enhances the accuracy and reliability of the data, leading to more robust and valid conclusions, which contribute to building a stronger body of evidence. Furthermore, standardized procedures also allow other researchers to replicate the study, which is a vital aspect of scientific research.

Bearing this in mind, we recently reported on the bioaccumulation of essential and potentially toxic trace elements in relation to the processing and location of the following five tea gardens: in Portugal, the Azores, Germany, Switzerland, and the Netherlands, where the sampling and processing techniques were standardized [7]. This systematic approach has now been extended to the present study, which was conducted on tea leaves of the same five tea gardens, processed to give green and black teas, and from which hot brews were prepared. The fundamental distinction between green and black teas lies in the degree of oxidation. Green tea is an unoxidized product where the leaves undergo heat enzyme inactivation (steaming or pan-firing) to prevent oxidation of the tea catechins. Black tea is instead fully oxidized; the leaves are withered, rolled, and subjected to fermentation/aeration, which allows the polyphenol oxidases and peroxidase enzymes to convert the simple tea catechins into larger molecules (theaflavins and thearubigins) that impart characteristic color and aroma. In our previous studies on European teas [4,5,6], green teas consistently exhibited significantly higher levels of total polyphenols (TPC) and flavonoids (TFC) compared to black teas, and this is directly correlated with their higher unoxidized catechin content. Specifically, green teas contained two to three times more TPC than their black counterparts grown in the same gardens. Interestingly, black teas exhibited higher metal chelating power than green teas, attributed to the presence of theaflavins and thearubigins, which possess specific chemical moieties (adjacent hydroxyl and carbonyl functions) that are highly effective at binding transition metal ions. Using ATR-FTIR spectroscopy, the green tea leaves displayed a distinct band at ~1340 cm^−1^, attributed to the C-O bond in catechins. This band is much lower or absent in black tea. Furthermore, hot black tea brews showed a unique band at ~1010 cm^−1^, representing the presence of quinoid oligomers (theaflavins/thearubigins) that are absent in green tea. The black teas generally displayed higher relative amounts of alkaloids (caffeine) with respect to green teas. UV-Vis spectroscopy revealed that green teas have a lower absorption in the 380–440 nm range, while the absorption intensity around 226 nm is highest and diminishes as the degree of oxidation increases, making it much lower in black tea.

In this systematic study, the teas were characterized in terms of the following: (a) antioxidant capacity using a panel of well-established assays; (b) metabolomic profiling using Ultra High Performance Liquid Chromatography–Mass Spectrometry (UHPLC-MS) for providing a comprehensive analysis of the various metabolites which contribute to tea’s flavor, aroma and health benefits; and (c) for the first time, sensory profiling of European teas, which could aid in assessing the overall quality of tea by comparing sensory attributes against established standards, and for providing information on consumer preferences and acceptance, which can guide product development and marketing strategies of European tea [8]. By employing this structured approach, the findings on antioxidant, polyphenolic, and sensory profiles are comparable, accurate, reliable, and meaningful. This ultimately advances knowledge in the field of tea research in Europe.

## 2. Materials and Methods

### 2.1. Chemicals and Equipment

All chemicals were purchased from Merck KGaA (Darmstadt, Germany): [2,2′-azinobis-(3-ethylbenzothiazoline-6-sulfonic acid) diammonium salt] (ABTS), 6-hydroxy-2,5,7,8-tetramethylchroman-2-carboxylic acid (TX), gallic acid (GA), Folin–Ciocalteu reagent (2N solution), potassium persulfate (K_2_S_2_O_8_), sodium carbonate (Na_2_CO_3_), 3′,6′-dihydrosyspiro[isobenzofuran-1[3H],9′[9H]-xanthen]-3-one (fluorescein), [2,2′-azobis(2-methylpropionamidine) dihydrochloride] (AAPH), iron(II) sulfate heptahydrate, (+)-Catechin hydrate, sodium nitrite (NaNO_2_), aluminum chloride hexahydrate (AlCl_3_·6H_2_O), sodium hydroxide (NaOH), 2,4,6-tripyridyl s-triazine (TPTZ), iron(III) chloride (FeCl_3_), ascorbic acid (AA), hydrochloric acid (HCl), potassium dihydrogen phosphate (KH_2_PO_4_), dipotassium hydrogen phosphate (K_2_HPO_4_), ethanol RPE grade. Ultrapure water was generated from a Milli-Q system by Merck Millipore (Merck KGaA, Darmstadt, Germany) and was used for all the experiments. Mineral water Acqua Sant’Anna S.p.A. (Vinadio, CN, Italy) with a fixed residue at 180 °C of 22 mg/L and total hardness of 0.98 °f used for tea infusions was purchased from the local supermarkets. Spectrophotometric VIS measurements were recorded on a microplate reader (Synergy HT, Biotek, Winooski, VT, USA). UHPLC analysis was performed on an Acquity H-class system equipped with a photodiode array detector (PDA) and mass spectrometer (MS) from Waters Corporation (Milford, MA, USA). The following standards for UHPLC analysis were purchased from Merck KGaA (Darmstadt, Germany): gallic acid (GA), chlorogenic acid (CGA), ellagic acid (EA), catechin (C), epigallocatechin gallate (EGCG), epicatechin gallate (ECG), kaempferol-glucoside, quercetin, and caffeine.

### 2.2. Tea Samples

**Figure 1:**
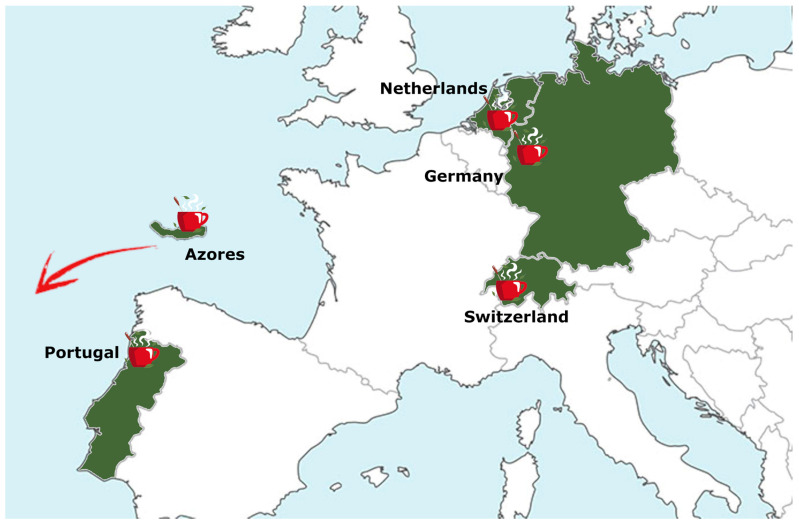
Graphical distribution across Europe of tea sample gardens (countries/islands shaded in dark green). The teacups indicate the location of the tea gardens.

Twelve tea samples *(Camellia sinensis)* were studied that were kindly provided by five tea gardens located across Europe (Figure 1): Het Zuyderblad (Netherlands), Casa del Tè Monte Verità (Switzerland), Tschanara Tea Garden (Germany), Chà Camèlia (Portugal), and Agrarian Development Services São Miguel, Azores (Portugal). Details regarding the temperature range of these gardens, location, growing season, altitude, humidity, and average rainfall can be found in the EuT Association leaflet and in Girolametti et al. [3,7]. Tea leaves were those of the first flush, harvested by hand (two leaves and a bud) between March and June 2023, based on the harvesting season of each garden, harmonizing the plucking period of all participating members to the first two weeks after the start of the new tea harvest season. The leaves were all processed in the same way according to the guidelines set in place for this study, which followed those of the Compendium for tea production in Europe [9]. Overall, six green teas (G) and six black teas (B) reflecting the type of tea traditionally produced in each tea garden were collected. The general processing steps are outlined as follows: black tea was produced by withering of the tea leaves (20–24 h), followed by hand/electric rolling (15 min^−1^ h), oxidation (4–8 h at 20–30 °C), and drying (2–5 h at 80–90 °C); green tea was produced by heat enzyme inactivation of the leaves (steaming or wok/pan-firing for 5–7 min at 250–300 °C), followed by hand/electric rolling (5–10 min) and drying (1–2 h at 80–120 °C). The tea samples are described in Table 1 where they are identified with an acronym of three letters indicating the country of origin: N = Netherlands, S = Switzerland, P = Portugal, A = Azores, G = Germany, followed by the type of tea: green = G; black = B; the third letter identifies a distinctive trait: for Portuguese tea (Chà Camelia), samples were obtained from plants propagated either from cuttings (C) or seeds (S); for Azores tea (São Miguel), the samples differ based on two distinct cultivation locations: Ribeira Grande (R) at an altitude of 50 m a.s.l. and Sete Cidades (S) at 266 m a.s.l.; for German tea (Tschanara Teagarden), the samples are distinguished by the type of cultivar: Korean (K) or Japanese (J).

### 2.3. Preparation of Tea Brews

The brews were prepared from 1.0 g of tea leaves previously ground using a STO 6509 hand grinder(SINBO, Istambul, Türkiye), to guarantee a homogeneous extraction surface for each of the samples, regardless of the type of leaf. Samples were infused in 50 mL of mineral water previously heated to 95–100 °C for 5 min and then filtered through Whatman No. 4 filter paper, aliquoted, and stored at −20 °C until analyzed [4,5,10]. Mineral water rather than tap water for the preparation of tea was chosen since its composition is stable and known. Each brew was prepared in triplicate for each sample on three separate days.

### 2.4. Determination of Total Phenolic Content (TPC)

Total phenolic content in the tea infusions was determined using the Folin–Ciocalteu method [4,11] adding to 50 μL of each diluted tea sample 30×, 150 μL of a 10-fold diluted aqueous solution of Folin–Ciocalteu reagent; after 10 min incubation in the dark at room temperature, 100 μL of a 10% *w*/*w* Na_2_CO_3_ aqueous solution was added and the samples were then incubated at room temperature in the dark for 120 min before measurement of absorbance at 760 nm against the same mixture containing water instead of tea sample as blank. The results are expressed as mM Gallic acid equivalents (mM GAEq), using the linear regression value calculated from a gallic acid calibration curve (final concentrations in water: 0.05–0.60 mM).

### 2.5. Determination of Total Flavonoid Content (TFC)

The total flavonoid content in the tea infusions was measured using a colorimetric assay according to the method of Kim et al. with some modifications [4,12]. Briefly, 50 μL of each tea infusion diluted 10× was added to each well of a transparent 96-well microplate containing 150 μL of water. The following solutions were then added in the order reported and at the defined times: 12 μL of 5% *w*/*w* NaNO_2_ solution; after 5 min, 12 μL of 10% *w*/*v* AlCl_3_ aqueous solution; after 1 min, 80 mL of 1 M NaOH aqueous solution. After 15 min of incubation at room temperature in the dark, the absorbance was read at 510 nm against water as a blank. The results are expressed as mM catechin equivalents (mM CEq), using the linear regression value calculated from a catechin calibration curve (final concentrations in water: 0.02–0.35 mM) that was run during the assay at the same time as the tea samples.

### 2.6. Determination of In Vitro Antioxidant Capacity (ABTS, FRAP, and ORAC)

The in vitro antioxidant capacity was evaluated by a panel of three different assays, namely, Oxygen Radical Absorbance Capacity (ORAC) [13], ABTS [14], and Ferric Reducing Antioxidant Power (FRAP) assays [15], and as previously reported with some modifications in [4].

#### 2.6.1. ORAC Assay

For the ORAC assay, in each well of a solid black 96-well microplate, 50 μL of each tea infusion diluted 600× with PBS (phosphate-buffered saline, 75 mM, pH 7.4) was mixed with 160 μL of a 0.010 µM solution of fluorescein in PBS. After 10 min of incubation in the dark at 37 °C, 90 μL of 25 mM AAPH solution in PBS was rapidly added to each well, and fluorescence was recorded on a microplate reader, from the top, every 120 s for 3.5 h using an excitation wavelength of 485/20 nm, an emission filter of 528/20 nm, and a constant temperature of 37 °C. The reaction kinetics were typical of classic fluorescence decay due to bleaching of fluorescein that was delayed in the presence of tea samples or of Trolox used as a standard. The AUC (area under the fluorescence decay curve) was automatically calculated by the analytical software Gen5 2.00.18 (Biotek, Winooski, VT, USA) integrated into the Synergy HT microplate reader (Biotek, Winooski, VT, USA). The net AUC for each standard/compound was obtained by subtracting the area of the control sample that lacked antioxidants. The antioxidant capacity is expressed as mM Trolox equivalents (mM TXEq), using the linear regression value calculated from the Trolox calibration curve (final concentrations in PBS: 0.01–0.08 mM) that was run during the assay at the same time as the tea samples.

#### 2.6.2. ABTS Assay

A stock solution of the colored radical cation (ABTS^•^^+^) was prepared by mixing a 7.0 mM aqueous ABTS solution with a 24.5 mM aqueous solution of potassium persulfate as the oxidizing agent in a 9:1 ratio, respectively, and allowing the mixture to stand at room temperature in the dark for 12–16 h before use. This stock solution was then diluted 50-fold with water to reach an absorbance of 0.9 ± 0.1 at 734 nm. For this assay, 30 μL of each brew previously diluted 120× with water was added to each well of a transparent 96-well microplate, followed by 270 μL of the diluted ABTS^•+^ solution. The microplate was shaken and left to stand for 120 min at room temperature in the dark before measuring the absorbance at 734 nm against water as a blank. The antioxidant capacity was determined as inhibition percentage and is expressed as mM Trolox equivalents (mM TXEq), using the linear regression value calculated from the Trolox calibration curve (final concentrations in water: 0.005–0.250 mM) that was run during the assay at the same time as the tea samples.

#### 2.6.3. FRAP Assay

For the FRAP assay, the working reagent was prepared by mixing immediately before usage the following reagents in a 5:5:50 ratio, respectively: 10 mM TPTZ solution in 40 mM HCl, 20 mM FeCl_3_ in water, and 300 mM acetate buffer, pH 3.6. This reagent (250 μL) was then added to 50 μL of each brew previously diluted 40X with water that was present in each well of a transparent 96-well microplate. The microplate was shaken and incubated for 30 min at room temperature in the dark before absorbance measurement at 600 nm. The results are expressed as mM ascorbic acid equivalents (mM AAEq), using the linear regression value calculated from the ascorbic acid calibration curve (final concentrations in water: 0.01–0.60 mM) that was run during the assay at the same time as the tea samples.

### 2.7. UHPLC-PDA-MS Analysis of Phenolic Compounds and Caffeine

Tea samples were filtered through a 0.45 µm regenerated cellulose filter (Sartorius, Goettingen, Germany) prior to injection into a UHPLC-PDA-MS system to analyze phenolic compounds. For caffeine determination, the samples were diluted 10× and analyzed following the same chromatographic conditions used for phenolic compounds. The chromatographic separation was performed on an Accucore C18 column (150 × 2.1 mm, 2.6 µm; Thermo Scientific, Waltham, MA, USA) set at 40 °C. The mobile phase consisted of water (A) and acetonitrile (B), both acidified at 0.1% (*v*/*v*) with formic acid, at a constant flow rate of 0.4 mL/min. The gradient started at 5% B for 1 min, from 5% to 70% B in 19 min, up to 95% B in 5 min, then kept in isocratic mode for 5 min at 95% B before restoring the initial conditions from 31 to 35 min with 5% B. PDA was set at 280 nm, and absorbance spectra were recorded from 200 to 500 nm. MS was operated in negative (for phenolic compounds) and positive (for caffeine) electrospray ionization mode, acquiring in full scan from 100 to 610 *m*/*z*. Cone voltage was set at 15 V, and capillary voltage was 0.8 kV. Data were acquired and processed using Empower 3.0 software (Waters, Milford, MA, USA).

Retention time, UV, and mass spectra were used to identify phenolic and alkaloid compounds. The quantification of compounds was performed by UHPLC-MS using a 5-point calibration curve obtained by diluting a stock solution of GA, C, CGA, EA, EGCG, ECG, quercetin, kaempferol–glucoside, and caffeine in the range of 1–500 ng injected in the column. Good linearity (R^2^ > 0.993) was recorded for all compounds. Other identified compounds were quantified based on available standards, as follows: GA was used as a reference for galloylquinic acid (GQA); C for gallocatechin (GC), epigallocatechin (EGC), and epicatechin (EC); EGCG for gallocatechin gallate (GCG); kaempferol–glucoside for quercitrin and astragalin; and quercetin for myricetin and kaempferol. Results are expressed as average and standard deviation in mg/L.

### 2.8. Sensory Analysis

A preference test was performed using a panel of six well-trained tea sommeliers, comprising five females and one male, whose ages ranged from 30 to 60 years. All participants volunteered to take part in the study. Participation was entirely voluntary, and no coercion or incentives were used. Prior to participation, all individuals were informed about the aims of the study, the experimental procedures, and the absence of foreseeable risks. Informed consent was obtained verbally from all participants before the experiment. Participants were informed that they could withdraw from the study at any time without any consequences. No personal or identifying information was collected, and all data were recorded and analyzed in anonymized form. The rights, privacy, and confidentiality of all participants were fully respected throughout the study. This study involved a non-invasive sensory experiment conducted with adult volunteers and did not pose any foreseeable risk to participants. Based on established institutional practice for similar sensory studies, ethical approval was not requested prior to the execution of the experiment. 

The tea infusions were prepared according to standard procedures for tea tasting. Briefly, for green teas, 2.5 g of leaves were brewed in 150 mL of water for 3 min at 85 °C, whilst for black teas, this was set at 95 °C. Tea infusions were labeled with a three-digit code and presented to each assessor in a randomized order, separating green samples from the black ones. Water was provided for palate cleansing between each sample tasting. During the tea tasting, each assessor used their own spoon to sample the tea and recorded their observations on a sensory evaluation sheet, which considered numerous aspects: color, aroma, and size of the infused and dry leaves; color, intensity, and reflections of the liquor in the cup; smell; flavor; texture; taste; mouth aromas; and final aftertaste. Additionally, they expressed an overall liking score for the teas, assigning ratings from 1 to 9 for the parameters of appearance, taste, and smell. These attributes were evaluated using a Hedonic 9-point scale (9 extremely like, 5 neutral, 1 extremely dislike) [16]. In addition to overall liking, the flavor (sweet, sour, bitter, and umami) and mouthfeel notes (cereal, phenolic, marine, herbaceous, floral, sugary, fruity, woody, and spicy) were considered, and in this case, the frequency of the attribute was rescaled on a percent proportion and used to plot a 100% stacked column chart. The results were statistically analyzed to show the acceptance of the teas evaluated [17]. The collected data were processed to generate radar charts and summary histograms of overall liking and sensory appreciation.

### 2.9. Statistical Analysis

The results of the TPC, TFC, ORAC, ABTS, and FRAP tests are expressed as mean values with standard deviation (SD) from at least three independent experiments, each performed on each of the three prepared infusions (*n* = 9). Statistical differences were obtained through an analysis of variance (ANOVA), followed by Tukey’s multiple comparison test at a 95% confidence level (*p* ≤ 0.05). These statistical treatments were performed using XLSTAT software v2018.1.1 (Addinsoft SARL, Paris, France) and Statistica v. 10 (StatSoft, Inc., Tibco, CA, USA). PCA and hierarchical clustering heatmaps were performed to visualize differences in the level of phenolic and alkaloid compounds using the online software Metaboanalyst version 6.0.

## 3. Results

### 3.1. Total Polyphenol and Flavonoid Contents

**Figure 2:**
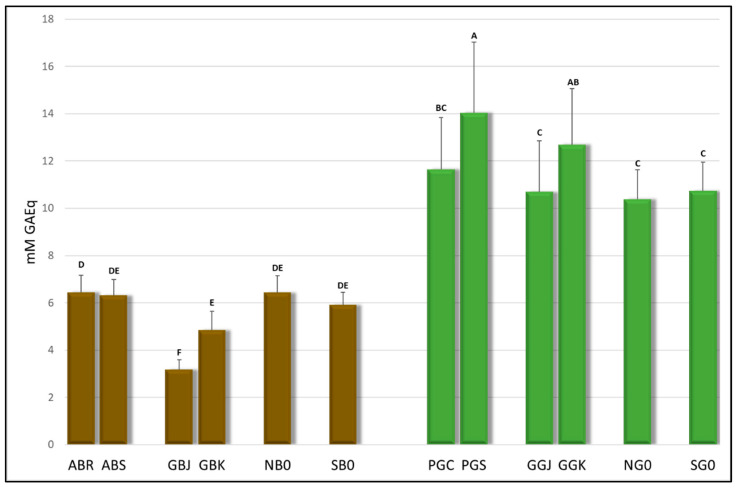
Total polyphenol content (TPC) of the tea brews measured using Folin–Ciocalteu’s reagent. Bars are colored according to the type of tea (brown = black tea; green = green tea). The letters above the bars indicate the homogeneous sub-classes resulting from Tukey’s post hoc multiple comparison tests (*p* < 0.05). ABR = Azores Black Ribeira Grande; ABS = Azores Black Sete Cidades; GBJ = Germany Black Japan; GBK = Germany Black Korea; NB0 = Netherlands Black; SB0 = Switzerland Black; PGC = Portugal Green Cuttings; PGS = Portugal Green Seeds; GGJ = Germany Green Japan; GGK = Germany Green Korea; NG0 = Netherlands Green; SG0 = Switzerland Green.

The total content of polyphenols (TPC) in the tea brews is reported in Table 2 and Figure 2, where the green teas (green bars) are plotted separately from the black ones (brown bars).

The results show the presence of a significantly higher polyphenol content in green teas (GGJ, GGK, NG0, and SG0), on average twice the amount, compared to the corresponding black teas (GBJ, GBK, NB0, and SB0), as already observed for infusions obtained from leaves coming from the same regions in different years [4]. The sample with the highest polyphenol content is the green tea from Portugal (PGS) with a TPC of 14.03 mM GAEq, and this result is consistent with that reported in [4]. Furthermore, comparing PGS, obtained by propagating the plant from seeds, with PGC, propagated by cuttings, a significantly higher polyphenol content is observed in the former (PGS). The black tea samples from the Azores (ABR and ABS) show no significant differences among each other regarding TPC, indicating that the 200 m difference in altitude of the two gardens where the tea is grown appears to have no influence on this functional tea component. For the German teas, a significantly higher TPC can be observed in the Korean variety (GBK, GGK) compared to the Japanese one (GBJ and GGJ).

The total flavonoid content reported in Table 2 was determined using the aluminum chloride method to separate the flavonoid contribution from that provided by the total polyphenols. The results show a similar trend to the polyphenol content. However, no significant differences were observed between teas from Portugal propagated in different ways (PGC and PGS), nor between the two German green teas obtained from the two different cultivars (GGJ and GGK), although the differences between these two cultivars are maintained in the black teas, with the Korean cultivar showing the highest amount (GBJ and GBK). Also in this analysis, the German black tea GBJ shows the lowest flavonoid content among all the teas.

### 3.2. Antioxidant Capacity

The antioxidant capacity of brews obtained from teas grown in Europe was evaluated using three different spectrophotometric assays in order to generate a more complete antioxidant profile of the different teas that better reflects their potential protective health effects, as follows: ORAC, FRAP, and ABTS [18]. The results obtained using these three assays are reported in Table 2.

The ORAC assay is the only direct method used in this study for the determination of antioxidant capacity since it describes the capacity of the studied matrix to inhibit the oxidative degradation of a fluorescent substrate induced by the constant generation of peroxyl radicals from a free radical generator.

The obtained results are expressed in mM TXEq and are reported in Table 2. Consistent with the results obtained in the polyphenol analyses, the green teas show a significantly higher antioxidant capacity with respect to black teas (24.8 mM TXEq vs. 14.1 mM TXEq). Overall, the Dutch green tea (NG0) has the lowest antioxidant capacity out of the green teas tested, while among the black teas, the one with the lowest is the German tea of the Japanese cultivar (GBJ). In addition, in all the tests performed comparing the antioxidant profile of all the black teas, GBJ stands out as being significantly different from all the rest. Interestingly, no significant differences were observed among the *assamica* variety of the Azores teas (ABR and ABS) compared to the *sinensis* variety of the remaining black teas, apart from GBJ. However, although the Japanese cultivar (GBJ) shows a lower antioxidant profile in the black tea category, for the green teas, the differences between the two German cultivars are not so stark: indeed, the antioxidant capacity of GGJ is similar to those of the green teas with the highest antioxidant levels. Among the green teas, in terms of antioxidant profile, they are all very similar except for the Dutch green tea, which consistently showed the lowest antioxidant capacity.

Another test used for the determination of antioxidant activity was the ABTS assay, which is considered an indirect method based on different mechanisms (hydrogen and/or single-electron transfer). Also in this case, the results reported in Table 2 are expressed as mM TXEq, and they follow a similar trend as the ORAC assay, with the only difference being that in this case, the Dutch (NG0) and Swiss (SG0) green teas show an antioxidant activity comparable to the other green teas. Among the black teas, those from the Azores (ABR and ABS) do not show statistically significant differences between each other nor with the Dutch and Swiss black teas (NB0 and SB0). The two German teas (GBJ and GBK) showed the lowest levels of antioxidant activity, with GBJ not only having the lowest levels compared to the Korean cultivar (GBK), but also compared to all the other teas, confirming the trend of the previous tests.

The FRAP assay is a method used to measure antioxidant capacity exclusively through an electron transfer process: the reduction in the ferric ion–TPTZ complex by antioxidants leads to an increase in absorbance at 600 nm, and the results are expressed in mM AAEq as reported in Table 2. As previously described for the other antioxidant capacity assays, the results for green teas are significantly higher compared to the black ones. In addition, comparing green and black teas within the same category, these do not show statistically significant differences. The only exception is the German black tea Japanese variety (GBJ) and the Dutch green tea (NG0), which show significantly lower levels in terms of antioxidant capacity compared to the other samples of the same type (black and green). Overall, the results obtained with the FRAP assay reflect the literature reports confirming that green teas have a greater antioxidant capacity than oxidized black teas [19,20,21,22].

The data obtained from the analyses with the different tests on tea infusions were also statistically analyzed to verify their correlation through the determination of the Pearson correlation coefficient (Table 3). The Pearson coefficients obtained show a significant linear correlation (*p* < 0.0001) between all the tests. In particular, all the phenolic classes (both polyphenols in general and flavonoids in particular) contribute to the antioxidant activity of the infusions, with correlation coefficients for the FRAP, ABTS, and ORAC tests greater than 0.90 (*p*< 0.0001).

### 3.3. Profiling of Phenolic Compounds and Caffeine

In the tea infusions, 1 alkaloid (caffeine) and 16 phenolic compounds were identified, including 7 catechins belonging to the sub-category of flavanols (GC, EGC, C, EC, EGCG, GCG, and ECG), 4 phenolic acids (GQA, GA, CGA, and EA), and 5 flavanols and their glycosylated derivatives (myricetin, quercetin, kaempferol, quercitrin, and astragalin) (Figure S1).

**Figure 3:**
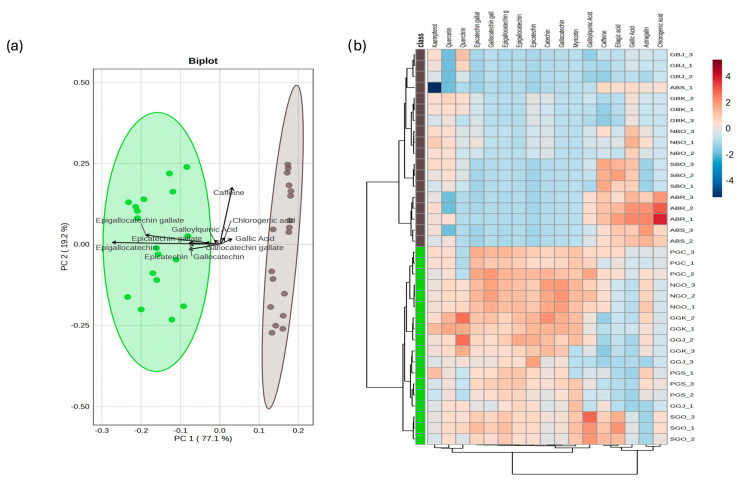
Differences between green tea (green) and black tea (gray). (**a**) PCA biplot of phenolic compounds and caffeine. (**b**) Hierarchical clustering heatmap; cell colors indicate low (blue) or high (red) concentration of identified compounds.

The PCA of green and black tea revealed that the first two PCs account for roughly 96% of the variance. In detail, PC1 (77%) separated black and green tea mainly based on differences in the concentration of catechins, while PC2 (19%) discriminated the samples based on caffeine content (Figure 3a). The hierarchical clustering heatmap clearly shows the abundance of catechins in all green tea samples, while phenolic acids (e.g., chlorogenic, gallic, and ellagic acid) are prevalent in black tea samples from the Azores (Figure 3b). Interestingly, the black tea samples are the only samples in this study of the *assamica* variety, which could account for the differences observed in the phenolic acids. The concentration of phenolic compounds in green tea is more than five times higher than in black tea (1345 mg/L vs. 248 mg/L, respectively, as average; see Table S1). Catechins accounted for 86–94% of total phenolic compounds in green tea, with EGC and EGCG being the predominant ones. Additionally, myricetin was detected only in green tea, even if at low concentrations.

Regarding caffeine, the type of processing (black vs. green) did not seem to affect its content as much as geographical origin and propagation method (cuttings vs. seeds). Indeed, tea samples from Germany displayed the lowest caffeine concentration with values varying from 275 to 349 mg/L in GBK and GGJ, respectively, whereas tea samples from gardens in the Azores and Switzerland, obtained from cuttings, showed the highest concentration of caffeine (604–686 mg/L).

### 3.4. Sensory Analysis Results

To explore the differences in flavor between the studied teas produced in different regions of Europe, to assess whether some specific characteristics of teas could influence the quality of the infusions, and to understand the consumers’ liking for them, we performed a traditional sensory evaluation.

**Figure 4:**
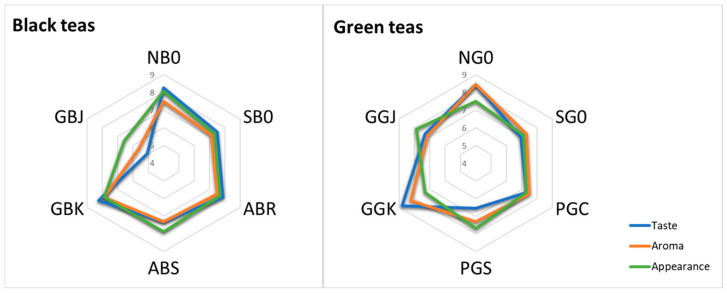
Radar graphs depicting the appreciation of green and black tea infusions obtained from the sensory analysis in terms of taste (blue line), aroma (orange line), and appearance (green line).

**Figure 5:**
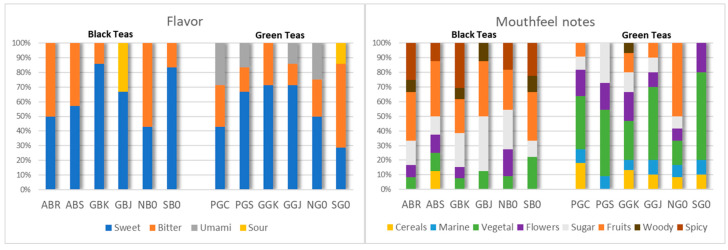
Flavor and mouthfeel notes perceived from the sensory analysis on black and green tea infusions reported as a percentage.

The sensory perceptions described by the tea tasters were processed to obtain the final data reported in Figure 4 and Figure 5. Regarding the overall tea appreciation, the tea tasters’ scores for sight, aroma, and taste were averaged, obtaining values ranging from 6.56 to 8.81 for green teas and from 5.06 to 8.25 for black teas. The results obtained are reported in Figure 4, where taste (blue line), aroma (orange line), and appearance (green line) are depicted with radar graphs, describing the appreciation of green and black infusions separately. Overall, the highest scores for taste and aroma were obtained from the Dutch teas (NBO-NGO) and German teas, Korean cultivar (GBK-GGK), which scored highest in both the green and black tea infusions. GGK scored highest among all the green teas for taste (8.81), while NG0 scored the highest for aroma (8.44).

Comparing the Portuguese green teas (PG) amongst each other, we see that PGC (propagated by cuttings) scores higher than PGS (propagated by seeds) in terms of taste. Portuguese green tea PGS scores lower than all the others for taste but scores similarly for appearance and aroma to PGC, SG0, and GGJ. German green tea Japanese variety (GGJ) scores highest of all for appearance of infusions (7.88), but lower than the others for aroma (7.13).

In terms of the taste of black teas, the Dutch tea NB0 and the German tea Korean variety GBK (8.25) are followed by the Azores black tea ABR (7.88), while in terms of aroma, the best black teas are GBK, with the highest value (7.88), followed by NB0 and ABR with the same score (7.50). Dutch tea NB0 (8.06) is the best for the appearance of the infusion, followed by ABS and GBK (7.88). When comparing the two Azorean teas from the locations of Ribeira Grande (ABR) and Sete Cidades (ABS), their values are very similar to each other; however, ABR, compared to ABS, fares slightly better in terms of taste and aroma, but not in terms of appearance. Finally, it is worth noting that of the two German teas (GB), GBK clearly excels in all aspects, while GBJ ranks last among all black teas.

The analysis of the sensory evaluation sheets, which encompassed all aspects of the teas, from the dry leaves to the final infusion, highlighted the perceived flavor profiles (sweet, sour, bitter, and umami) and the nuanced palate notes, including cereals, marine, vegetal, floral, sugary, fruity, woody, and spicy characteristics. For each flavor and aroma perceived by each taster, a point was assigned. The total score for each tea was then calculated by summing the points awarded for each individual flavor and aroma. These results are reported in Figure 5, where the characteristics of each tea are reported as a percentage.

Concerning flavor (sweet, bitter, umami, and sour), black teas show a predominant sweet component except for NBO, where a bitter taste was predominant. The bitter taste was lowest in GBK and SBO; the only black tea that showed a sour component was GBJ. Regarding green tea, the umami flavor was perceived in most of them (PGC, PGS, GGJ, NG0).

The analysis of the mouthfeel notes of the black teas shows a predominant fruity component, followed by a spicy and sugary aroma, which in some cases (GBJ and NB0) could considerably impact the overall flavor. In particular, it can be noted that GBJ is the only one that lacks the spicy aroma. Noteworthy is also the presence of the woody aroma in some black teas (ABR, GBK, GBJ, and SB0) and the cereal aroma in the Azorean black tea ABS.

Green teas exhibit a predominantly vegetal aroma, complemented by notable floral, fruity, cereal, and marine notes, the latter two being generally absent in black teas. The Dutch green tea NG0, the tea taster’s favorite, shows a predominant fruity component compared to other green teas.

## 4. Discussion

This study was conducted with the understanding that Europe’s unique climatic and soil conditions can produce teas with distinct flavor profiles and chemical compositions, and this diversity enriches the global tea market by introducing new and unique tea experiences. The results presented here, using a systematic and comparative approach, show that there are indeed several differences among the teas grown across the European territory, not only from the antioxidant profile but also from the metabolomic and sensory profiles.

Firstly, the polyphenol profile and antioxidant capacity of teas from the five European gardens are comparable to those cultivated in other regions globally, as previously reported, despite the high variability in assay conditions and brewing techniques across the numerous studies examined [4,23]. The strong differences highlighted between the green and black tea are mainly related to the processing method, which, in the case of black tea, involves an oxidation step that causes the degradation of most of the phenolic compounds, especially catechins [24], into the higher molecular weight compounds theaflavins and thearubigins that impart the dark color, aroma, and flavor of black tea. Catechins are potent antioxidants, and their role in the prevention and protection against oxidative stress-related diseases, such as cardiovascular diseases and cancer, has been widely investigated [25,26]. Besides catechins, the antioxidant capacity of the tea infusions is enhanced by the high concentration of caffeine [27], which has been compared here for the first time in European teas. Caffeine, also referred to as theine in tea, is an alkaloid responsible for the increase in alertness and reduced fatigue associated with drinking tea. Interestingly, the tea samples from Germany propagated by seeds displayed the lowest caffeine concentration, whereas tea samples from the gardens in the Azores and Switzerland, obtained from cuttings, showed the highest concentration of caffeine. This variability in caffeine could be due to the propagation method, although there is no direct evidence in the literature to confirm this. It is well known that seed propagation leads to greater genetic diversity among tea plants, which can result in significant variations in polyphenol content and other chemical compounds. This is often beneficial for breeding programs and for developing new tea varieties with unique characteristics, but when more uniform and consistent chemical profiling, including polyphenol content, is desired, such as for commercial tea production, then propagating by cuttings generating clonal tea cultivars is preferred. Furthermore, cuttings generally result in faster growth and earlier maturity compared to seed-propagated plants, which are beneficial for commercial tea plantations aiming for quicker harvests [28,29,30]. It is unlikely that the differences among the caffeine contents are related to the two different varieties of tea, *assamica* vs. *sinensis*, since Azores teas are from the variety *assamica,* whereas the Swiss teas and German teas are both the *sinensis* variety. This is in contrast with a recent study comparing caffeine content in *assamica* and *sinensis* varieties, where *assamica* ones showed significantly lower caffeine accumulation [31]. Notable differences were, however, observed based on the propagation method for the tea samples from the same tea garden—Chà Camèlia in Portugal. Although the samples were all derived from the same variety (*Camellia sinensis sinensis*) and processed identically as green tea, the tea propagated by seeds (PGS) exhibited a significantly higher total polyphenol content compared to that propagated by cuttings (PGC). However, this variation in polyphenol levels did not translate into differences in antioxidant capacity since both propagation methods led to comparable results across the panel of antioxidant assays. The variation in caffeine content in these two teas (PGC > PGS) appears to have only a minor influence on their overall antioxidant capacities. Firstly caffeine is less potent than catechins as free radical scavenger and metal chelator and secondly, the assays employed in this study to assess antioxidant capacity primarily measure the hydrogen-donating or reducing potential of antioxidant compounds, whereas caffeine is believed to exert its antioxidant effects in vivo mainly via alternative mechanisms, such as through the purinergic signaling pathway and glutamatergic neurotransmission, that are not detectable by the analytical methods used in this study [27,32].

It is well known that the unique flavors and aromas of different types of tea, each with its wide variety of compounds imparting specific sensory characteristics like umami, astringency, sweetness, and fruity or floral notes, may correlate with specific chemical profiles. This in turn depends on environmental and processing conditions, such as oxidation, roasting, and drying which could all play important roles in shaping the sensory profile of tea [33]. In general, in tea infusions, flavonol-O-glycosides, tannins and galloylated catechins are the main astringent compounds, while bitterness is enhanced by the presence of caffeine and non-galloylated catechins. Furthermore, theanine, succinic acid, gallic acid and theogallin contribute to the umami taste while sweetness is a unique perception of green tea, attributed to the hydrolysis of galloylated catechins [34]. Hence, it is not surprising that the sensory analysis performed for the first time on these European teas revealed noteworthy and informative findings. However, a direct correspondence between chemical composition and perceived sensory attributes could not be established, highlighting a methodological limitation of this study, since chemical and sensory analyses were conducted under slightly different infusion conditions. For this reason, the observed associations outlined below represent general trends rather than direct mechanistic links.

Caffeine is known to be associated with a more bitter taste; however, the teas that appeared to reveal a stronger bitterness, such as NBO, surprisingly had a low caffeine content compared to SGO and the two black teas from the Azores, which were also found to be bitter but had a high caffeine content. Concerning the overall appreciation of the tea infusions, the results appear to indicate that there is a negative correlation with the polyphenol content or antioxidant capacity. Seemingly, among the black teas, GBK was the one most highly appreciated in terms of taste, aroma, and appearance, although it exhibits one of the lowest TPCs, a finding similar to that of the green tea from the Netherlands (NGO), which has the lowest TPC among the green teas and the lowest antioxidant capacity, but is the one most highly appreciated for its taste, appearance, and aroma. This appears to be in accordance with the literature and our previous findings, which showed that African and Chinese white teas had a lower polyphenol content than green teas from the same countries but were more palatable and less bitter because of this [34,35]. Furthermore, if leaves are milled, which increases the surface area for polyphenol extraction into the infusion, they result in a more bitter infusion than infusions made from whole leaves [35]. However, the other German black tea studied from a Japanese cultivar (GBJ), which has overall the lowest content of all polyphenols examined (Figure 3b), ranked last in terms of appreciation for its taste, aroma, and appearance, which might be linked to this particular cultivar. Apparently, the cultivar stands out as one of the most crucial genetic properties for the tea plant, which could contribute to the unique metabolism that may ultimately affect the sensory profile [36]. On comparing the polyphenolic profile of GBJ with GBK (Figure 3b), it can be noted that quercetin, gallic acid, epicatechin, and epicatechin gallate are higher in the latter cultivar, with only quercitrin resulting in the highest in GBJ. Conversely, the Portuguese green tea propagated from seeds (PGS), which has the highest TPC among all teas in this study and high antioxidant capacity, was the least appreciated among the green teas for its taste. Based on this overall analysis, it can be concluded that while some European teas appear to offer fewer beneficial effects in terms of antioxidant capacity and total polyphenol content, they may nonetheless be more widely consumed and appreciated for their flavor and sensory qualities, contributing to increasing their market value. Although a detailed analysis of volatile compounds, which play a crucial role in defining the sensory characteristics and economic value of tea [37], was not conducted, this comparative approach has nevertheless yielded valuable insights.

The European tea landscape represented by the five tea gardens considered in this study encompasses a wide diversity of microclimatic conditions and growing environments, with harvest periods typically extending from March to October. These heterogeneous conditions are expected to shape tea plant metabolism in ways that directly influence the chemical composition and sensory characteristics of the final product, beyond the effects attributable to processing alone. The regions studied differ substantially in altitude, ranging from 29 m a.s.l. in the Netherlands to 320 m a.s.l. in Switzerland, as well as in temperature regimes, with Germany exhibiting the largest annual thermal amplitude (−13 to 38 °C) and the Azores the most buffered climate (4.9–28 °C). Marked differences are also observed in humidity and average annual rainfall, from approximately 86% relative humidity and 1600 mm year^−1^ in the Azores to 40–70% and ~850 mm year^−1^ in the Netherlands, respectively [3].

Altitude has been widely reported to influence tea phenolic composition through the combined effects of climatic stressors (e.g., temperature and precipitation), modulation of growth rates, and soil–microbial interactions, resulting in measurable changes in total polyphenol content (TPC), individual catechin profiles, and antioxidant capacity. However, these effects are not linear and vary considerably with region, cultivar, and associated environmental variables [38]. For example, within the Gorreana plantations of the Azores, teas cultivated at moderate elevations (~300–350 m a.s.l.) have been reported to exhibit higher total phenolics, individual catechins, and antioxidant capacity compared with lower-lying plots from the same estate, highlighting the influence of altitude even across relatively modest elevation gradients [39]. In contrast, the present study detected no marked differences in TPC or antioxidant activity between the two black teas produced at different elevations within the Agrarian Development Services of the Azores, reinforcing the non-linear nature of altitude–phenolic relationships. Comparable inconsistencies have been reported elsewhere; for instance, an inverse correlation between altitude and TPC was observed in Chinese green teas [40]. More broadly, the chemical characteristics within both black and green tea categories were largely comparable across the five European locations studied, despite substantial differences in altitude, temperature range, rainfall, and humidity. One possible explanation for this relative compositional uniformity is the specific genetic background of *Camellia sinensis* and *C. sinensis* (*assamica*) varieties cultivated in Europe, which have undergone long-term selection through natural pressures such as frost exposure, wind desiccation, and delayed spring growth. Such adaptation may attenuate the magnitude of environmental effects typically observed in more conventional tea-growing regions, thereby contributing to the overall convergence of chemical traits observed in this study. The comparatively lower TPC, TFC, and antioxidant capacity measured in the Japanese cultivar of the German black tea (GBJ) are therefore most plausibly attributable to cultivar-specific characteristics. By contrast, the Dutch green tea (NG0), which exhibited the lowest antioxidant capacity, TFC, and TPC among the green teas, may reflect a stronger influence of local microclimatic conditions. This tea garden is the lowest-lying site evaluated, characterized by comparatively low humidity and rainfall and a maximum recorded temperature of 25 °C relative to the other green tea-producing locations. Interestingly, despite its lower phenolic content and antioxidant capacity, NG0 received high panel ratings for aroma and appearance in sensory evaluation. Together with the strong sensory appreciation of the German teas (GBK-GGK), these findings diverge from the canonical paradigm linking altitude-driven modulation of polyphenol composition to infusions with reduced bitterness and astringency, increased freshness, and enhanced aromatic complexity [41]. Collectively, these results underscore the multifaceted and non-linear influence of microclimate, location, and plant genetic background on chemical quality, antioxidant potential, and sensory identity. Such interactions must be carefully considered when interpreting compositional and sensory differences in teas cultivated under non-conventional growing conditions, such as those encountered in emerging European tea-producing regions.

Finally, this study focuses on European teas and provides valuable insights into a relatively unexplored area. The findings may assist tea growers in making informed decisions regarding cultivar selection, varietal choice, and propagation methods that could enhance the marketability of their teas in alignment with consumer preferences and market demands. Regarding this latter aspect, there is a growing interest in locally produced and sustainable products, and although European tea producers have the potential to meet the growing demand for sustainable and locally sourced products, the current scale of production remains insufficient. Although European teas may currently lag behind traditional tea-exporting countries in terms of global recognition and competitiveness, further investment and research could enable European tea agriculture to thrive, ultimately contributing to a more sustainable and diversified supply chain.

## 5. Conclusions

The understanding developed from this study offers a structured characterization of teas grown in five European gardens, confirming that green teas consistently possess higher total phenolic content (TPC), flavonoid content, and antioxidant capacity compared to black teas. Metabolomic analysis showed that while processing distinguishes green from black tea, caffeine levels are primarily influenced by geographical origin and propagation methods, such as the use of cuttings vs. seeds. Significantly, the research established a negative correlation between high TPC and sensory appreciation, as the teas most preferred by expert tasters, such as the Dutch and German (Korean cultivar) varieties, often displayed lower phenolic concentrations. These insights could aid European producers in better understanding the interplay between microclimate, genetics, and flavor, aiding in the selection of cultivars that align with market preferences for less bitter and more aromatic specialty teas.

## Figures and Tables

**Table 1:** General information and labeling of tea samples investigated.

European Garden *(Country)*	Processing Type	Variety	Cultivar	Propagation Method	Label
Het Zuyderblad (*Netherlands)*	Black	*sinensis*	unknown	Seeds	NB0
	Green	*sinensis*	unknown	Seeds	NG0
Tschanara Teagarden *(Germany)*	Black	*sinensis*	Korea	Seeds	GBK
	Green	*sinensis*	Korea	Seeds	GGK
	Black	*sinensis*	Japan	Seeds	GBJ
	Green	*sinensis*	Japan	Seeds	GGJ
Casa del Tè Monte Verità *(Switzerland)*	Black	*sinensis*	unknown	Cuttings	SB0
	Green	*sinensis*	unknown	Cuttings	SG0
Chà Camèlia *(Portugal)*	Green	*sinensis*	unknown	Cuttings	PGC
	Green	*sinensis*	unknown	Seeds	PGS
Agrarian Devt. Services	Black (Ribeira grande)	*assamica*	unknown	Cuttings	ABR
São Miguel	Black (Sete Cidades)	*assamica*	unknown	Cuttings	ABS
*(Azores, Portugal)*					

**Table 2:** Total phenolic content (TPC), total flavonoid content (TFC), and antioxidant activity data (ORAC, ABTS, FRAP) of the studied teas. Samples are grouped by type of tea (black or green), and the means are reported in bold. Letters within each column indicate homogeneous subclasses resulting from Tukey’s post hoc multiple comparison test (*p* < 0.05) performed between all samples.

	TPC (mM GAEq)				TFC (mM CEq)				ORAC (mM TXEq)				ABTS (mM TXEq)				FRAP (mM AAEq)			
ABR	6.43	±	0.73	D	0.85	±	0.13	DE	13.3	±	2.6	E	16.4	±	1.1	D	9.4	±	1.2	C
ABS	6.30	±	0.70	DE	0.87	±	0.17	DE	15.6	±	3.5	E	16.7	±	0.7	D	7.7	±	0.6	C
GBJ	3.18	±	0.41	F	0.41	±	0.10	F	8.8	±	2.5	F	10.1	±	1.3	F	3.6	±	0.3	D
GBK	4.84	±	0.81	E	0.83	±	0.08	DE	16.5	±	2.1	DE	13.6	±	0.8	E	9.5	±	3.0	C
NB0	6.42	±	0.73	DE	1.00	±	0.24	D	14.3	±	2.2	E	17.9	±	2.1	D	9.3	±	0.6	C
SB0	5.89	±	0.55	DE	0.71	±	0.09	E	15.8	±	3.0	E	16.4	±	1.2	D	8.9	±	0.6	C
***Black***	***5.51***	***±***	***0.65***		***0.78***	***±***	***0.14***		***14.1***	***±***	***2.6***		***15.2***	***±***	***1.2***		***8.1***	***±***	***1.1***	
PGC	11.64	±	2.20	BC	1.64	±	0.19	B	24.3	±	2.9	ABC	29.5	±	1.7	A	24.3	±	2.8	A
PGS	14.03	±	3.01	A	1.68	±	0.13	AB	28.5	±	3.5	A	29.2	±	2.0	AB	22.6	±	4.5	A
GGJ	10.68	±	2.16	C	1.79	±	0.18	AB	23.3	±	6.0	BC	27.5	±	1.2	BC	22.4	±	2.8	A
GGK	12.69	±	2.38	AB	1.86	±	0.33	A	27.2	±	3.9	AB	27.6	±	1.9	BC	23.0	±	3.7	A
NG0	10.38	±	1.24	C	1.34	±	0.12	C	20.2	±	1.5	CD	26.7	±	1.5	C	18.2	±	3.4	B
SG0	10.72	±	1.23	C	1.37	±	0.12	C	25.1	±	4.9	AB	29.5	±	1.3	A	23.9	±	2.8	A
***Green***	***11.69***	***±***	***2.04***		***1.61***	***±***	***0.18***		***24.8***	***±***	***3.8***		***28.3***	***±***	***1.6***		***22.4***	***±***	***3.4***	

**Table 3:** Matrix of Pearson’s correlation coefficients (values are all different from 0 with a significance level alpha = 0.05). *p* values are all <0.0001.

Variables	TPC	TFC	ABTS	FRAP	ORAC
TPC	1	0.949	0.965	0.949	0.961
TFC	0.949	1	0.934	0.947	0.932
ABTS	0.965	0.934	1	0.979	0.934
FRAP	0.949	0.947	0.979	1	0.954
ORAC	0.961	0.932	0.934	0.954	1

## Data Availability

No data were used for the research described in the article.

## Supplementary Materials

The following supporting information can be downloaded at: https://www.mdpi.com/article/10.3390/antiox15010141/s1. Figure S1: Chromatographic profiles acquired at 280 nm of green (a) and black (b) tea from the Netherlands. 1—Galloylquinic Acid, 2—Gallic Acid, 3—Gallocatechin, 4—Epigallocatechin, 5—Catechin, 6—Chlorogenic acid, 7—Caffeine, 8—Epicatechin, 9—Epigallocatechin gallate, 10—Gallocatechin gallate, 11—Ellagic acid, 12—Epicatechin gallate, 13—Quercitrin, 14—Astragalin, 15—Myricetin, 16—Quercetin, 17—Kaempferol; Table S1: Contents of phenolic compounds and caffeine in green and black tea sample. Results are expressed as average ± standard deviation in mg/L.
