# Supplementary material for: European Teas (Camellia sinensis) as a New Frontier in the Specialty Tea Market: Characterizing the Antioxidant, Polyphenolic, and Sensory Profiles Through a Systematic, Comparative Approach
Source: Antioxidants (Basel). 2026 Jan 22;15(1):141. doi: 10.3390/antiox15010141 (PMC12837149; doi:10.3390/antiox15010141)
Supplement: Supplementary file 1 [file antioxidants-15-00141-s001.zip › antioxidants-4074352-supplementary.pdf]

SUPPLEMENTARY MATERIAL

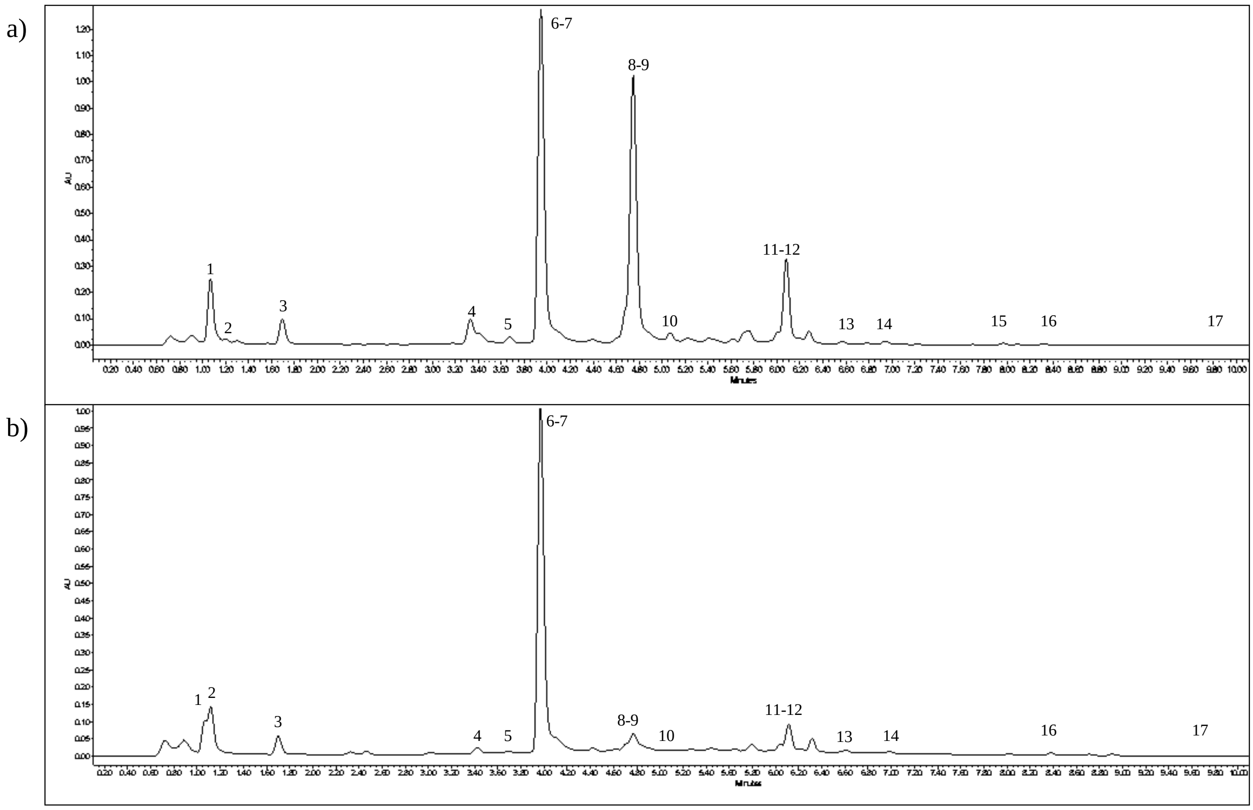

Figure S1. Chromatographic profiles acquired at 280 nm of green (a) and black (b) tea from Netherlands. 1-Galloylquinic Acid, 2-Gallic Acid, 3-Gallocatechin, 4-Epigallocatechin, 5-Catechin, 6-Chlorogenic acid, 7-Caffeine, 8-Epicatechin, 9-Epigallocatechin gallate, 10-Gallocatechin gallate, 11-Ellagic acid, 12-Epicatechin gallate, 13-Quercitrin, 14-Astragalin, 15-Myricetin, 16-Quercetin, 17-Kaempferol.

Table S1. Contents of phenolic compounds and caffeine in green and black tea sample. Results are expressed as average ± standard deviation in mg/L.

	GQA	GA	GC	EGC	C	CGA	EC	EGCG	GCG
[M-H]$^-$	343	169	305	305	289	353	289	457	457
RT	1.0	1.1	1.7	3.3	3.6	3.9	4.6	4.7	5.0
ABR	47.7 ± 0.6 ^{cde}	107.7 ± 15.1 ^{f}	-	1.4 ± 2.5 ^{a}	0.5 ± 0.7 ^{a}	136.2 ± 34.7 ^{c}	6.8 ± 2.5 ^{ab}	13.4 ± 6.7 ^{a}	-
ABS	32.3 ± 4.9 ^{abc}	74.4 ± 9.3 ^{e}	-	-	-	46.9 ± 12.2 ^{b}	2.2 ± 1.7 ^{a}	5.5 ± 0.8 ^{a}	-
GBJ	18.9 ± 2.9 ^{a}	32.0 ± 8.9 ^{bcd}	-	-	-	2.7 ± 0.5 ^{a}	7.2 ± 3.6 ^{ab}	4.2 ± 0.1 ^{a}	4.1 ± 0.01 ^{a}
GBK	26.4 ± 3.8 ^{ab}	48.4 ± 3.3 ^{d}	3.5 ± 1.3 ^{a}	13.8 ± 8.2 ^{a}	12.9 ± 2.4 ^{ab}	3.7 ± 1.8 ^{a}	67.8 ± 4.5 ^{cd}	23.7 ± 8.9 ^{a}	4.1 ± 0.1 ^{a}
NBO	32.5 ± 3.2 ^{abc}	84.3 ± 10.9 ^{e}	4.6 ± 0.9 ^{a}	20.1 ± 2.3 ^{a}	15.5 ± 1.8 ^{ab}	5.1 ± 0.8 ^{a}	54.7 ± 3.5 ^{bc}	47.9 ± 6.2 ^{a}	5.7 ± 0.4 ^{a}
SBO	27.8 ± 5.4 ^{ab}	78.5 ± 5.1 ^{e}	-	4.9 ± 1.5 ^{a}	-	3.6 ± 1.6 ^{a}	2.9 ± 0.8 ^{ab}	34.0 ± 0.9 ^{a}	-
PGC	59.8 ± 11.0 ^{ed}	35.1 ± 2.3 ^{cd}	116.4 ± 14.1 ^{cd}	457.8 ± 56.5 ^{b}	44.4 ± 10.6 ^{cd}	19.9 ± 5.0 ^{ab}	142.7 ± 24.2 ^{ef}	360.9 ± 30.4 ^{c}	73.3 ± 15.0 ^{cd}
PGS	35.9 ± 4.8 ^{abc}	8.9 ± 3.2 ^{a}	89.9 ± 14.3 ^{bc}	417.5 ± 42.6 ^{b}	25.3 ± 5.1 ^{bc}	19.7 ± 6.1 ^{ab}	107.6 ± 18.5 ^{de}	308.1 ± 23.2 ^{bc}	46.9 ± 8.4 ^{b}
GGJ	34.1 ± 1.4 ^{abc}	10.9 ± 4.4 ^{ab}	79.3 ± 25.0 ^{b}	415.1 ± 40.6 ^{b}	29.3 ± 19.0 ^{bc}	6.5 ± 2.6 ^{a}	165.6 ± 50.0 ^{f}	254.8 ± 48.8 ^{b}	32.5 ± 11.7 ^{b}
GGK	40.8 ± 2.8 ^{bcd}	21.5 ± 9.1 ^{abc}	144.3 ± 19.7 ^{d}	426.6 ± 53.9 ^{b}	60.7 ± 9.9 ^{d}	10.4 ± 2.2 ^{a}	165.7 ± 14.3 ^{f}	260.8 ± 29.7 ^{b}	53.5 ± 11.6 ^{bc}
NGO	57.8 ± 5.7 ^{ed}	20.7 ± 1.7 ^{abc}	181.8 ± 5.4 ^{e}	480.6 ± 12.6 ^{b}	63.7 ± 2.7 ^{d}	11.8 ± 1.1 ^{a}	147.6 ± 4.0 ^{ef}	359.3 ± 6.1 ^{c}	83.1 ± 4.8 ^{d}
SGO	83.1 ± 7.6 ^{f}	38.4 ± 2.9 ^{cd}	63.5 ± 8.4 ^{b}	338.3 ± 28.4 ^{b}	22.0 ± 4.0 ^{b}	34.8 ± 7.5 ^{ab}	116.2 ± 8.6 ^{def}	331.1 ± 20.6 ^{c}	49.3 ± 8.4 ^{b}

Continued

	EA	ECG	Quercitrin	Astragalin	Myricetin	Quercetin	Kaempferol	*Tot Phenols*	Caffeine
[M-H]$^-$/[M+H]$^+$ǂ	301	441	447	447	317	301	285		195ǂ
RT	5.9	6.0	6.5	6.7	7.2	8.5	9.8		4.0
ABR	17.1 ± 4.0 ^{cd}	22.8 ± 6.5 ^{a}	0.04 ± 0.02 ^{a}	6.3 ± 1.6 ^{e}	-	0.4 ± 0.6 ^{ab}	1.1 ± 0.0	*361.3 ± 73.4* ^{a}	609.1±8.0 ^{ef}
ABS	14.5 ± 1.9 ^{cd}	11.2 ± 2.7 ^{a}	-	5.1 ± 1.9 ^{de}	-	0.3 ± 0.6 ^{a}	0.7 ± 0.6	*193.3 ± 34.8* ^{a}	526.1 ± 18.2 ^{de}
GBJ	6.1 ± 0.3 ^{a}	14.8 ± 2.9 ^{a}	2.2 ± 1.2 ^{abc}	1.1 ± 0.7 ^{ab}	-	-	1.1 ± 0.1	*94.4 ± 18.9* ^{a}	300.9 ± 48.2 ^{ab}
GBK	5.6 ± 0.6 ^{a}	68.8 ± 6.6 ^{b}	1.4 ± 1.0 ^{ab}	1.5 ± 0.2 ^{abc}	-	1.1 ± 0.1 ^{bc}	1.1 ± 0.1	*283.7 ± 38.0* ^{a}	274.5 ± 34.8 ^{a}
NBO	8.0 ± 1.2 ^{ab}	79.2 ± 14.7 ^{bc}	1.0 ± 0.3 ^{ab}	2.6 ± 0.7 ^{abcd}	-	1.1 ± 0.0 ^{abc}	1.2 ±0.1	*363.5 ± 38.6* ^{a}	434.5 ± 71.8 ^{bcd}
SBO	18.1 ± 2.9 ^{d}	18.9 ± 2.8 ^{a}	0.9 ± 0.3 ^{ab}	-	-	1.1 ± 0.1 ^{abc}	1.1 ± 0.1	*191.8 ± 18.3* ^{a}	685.5 ± 11.1 ^{f}
PGC	12.4 ± 1.5 ^{bc}	193.4 ± 15.4 ^{g}	0.1 ± 0.1 ^{a}	2.4 ± 0.9 ^{abcd}	1.0 ± 0.0 ^{a}	1.1 ± 0.0 ^{bc}	1.1 ±0.1	*1522.0 ± 178. 4* ^{cd}	487.4 ± 79.2 ^{cde}
PGS	5.5 ± 0.4 ^{a}	120.7 ± 15.2 ^{cde}	0.4 ± 0.2 ^{a}	3.6 ± 1.0 ^{bcde}	0.7 ± 0.6 ^{a}	1.0 ± 0.0 ^{abc}	1.1 ± 0.2	*1192.8 ± 131.6* ^{bc}	386.6 ± 37.4 ^{abcd}
GGJ	5.9 ± 0.9 ^{a}	106.6 ± 27.8 ^{bcd}	3.1 ± 2.1 ^{bc}	1.2 ± 1.8 ^{ab}	0.7 ± 0.6 ^{a}	1.1 ± 0.1 ^{abc}	1.0 ±0.1	*1147.8 ± 303.0* ^{b}	349.3 ± 108.8 ^{abc}
GGK	7.0 ± 0.7 ^{a}	143.9 ± 23.9 ^{def}	4.5 ± 0.9 ^{c}	3.3 ± 0.8 ^{bccde}	0.8 ± 0.7 ^{a}	1.5 ± 0.2 ^{c}	1.2 ± 0.1	*1346.4 ± 170.7* ^{bcd}	281.3 ± 53.4 ^{ab}
NGO	7.8 ± 0.2 ^{ab}	178.1 ± 6.9 ^{fg}	2.3 ± 0.3 ^{abc}	4.3 ± 0.4 ^{cde}	1.1 ± 0.0 ^{a}	1.2 ± 0.0 ^{c}	1.1 ±0.1	*1602.4 ± 15.8* ^{d}	508.8 ± 17.0 ^{de}
SGO	19.6 ± 2.2 ^{d}	155.3 ± 19.5 ^{efg}	1.9 ± 0.7 ^{ab}	0.2 ± 0.1 ^{a}	1.0 ± 0.0 ^{a}	1.1 ± 0.0 ^{c}	1.0 ±0.1	*1256.8 ± 105.8* ^{bcd}	604.3 ± 36.9 ^{ef}

RT: retention time. GQA: galloylquinic acid; GA: gallic acid; GC: gallocatechin; EGC: epigallocatechin; C: catechin; CGA: chlorogenic acid; EC: epicatechin; EGCG: epigallocatechin gallate; GCG: gallocatechin gallate; EA: ellagic acid; ECG: epicatechin gallate. Different letters indicate statistical differences ($p < 0.05$).
